# The Neural Correlates of Mental Rotation Abilities in Cannabis-Abusing Patients with Schizophrenia: An fMRI Study

**DOI:** 10.1155/2013/543842

**Published:** 2013-07-17

**Authors:** Stéphane Potvin, Josiane Bourque, Myriam Durand, Olivier Lipp, Pierre Lalonde, Emmanuel Stip, Sylvain Grignon, Adrianna Mendrek

**Affiliations:** ^1^Centre de Recherche de l'Institut Universitaire de Santé Mentale de Montréal, 7331 Hochelaga, Montreal, QC, Canada H1N 3V2; ^2^Department of Psychiatry, Faculty of Medicine, University of Montreal, 2900 Edouard-Montpetit, Montreal, QC, Canada H3C 3J7; ^3^Department of Psychiatry, Faculty of Medicine, University of Sherbrooke, 580 Bowen Sud, Sherbrooke, QC, Canada J1G 2E8; ^4^Centre Hospitalier de l'Université de Montréal, 1058 St-Denis, Montreal, QC, Canada H2X 3J4; ^5^Department of Physiology, Faculty of Medicine, University of Sherbrooke, 3001 12e Avenue Nord, Sherbrooke, QC, Canada J1H 5N4; ^6^Department of Psychology, Bishop's University, 2600 College, Sherbrooke, QC, Canada J1M 1Z7

## Abstract

Growing evidence suggests that cannabis abuse/dependence is paradoxically associated with better cognition in schizophrenia. Accordingly, we performed a functional magnetic resonance imaging (fMRI) study of visuospatial abilities in 14 schizophrenia patients with cannabis abuse (DD), 14 nonabusing schizophrenia patients (SCZ), and 21 healthy controls (HCs). Participants performed a mental rotation task while being scanned. There were no significant differences in the number of mistakes between schizophrenia groups, and both made more mistakes on the mental rotation task than HC. Relative to HC, SCZ had increased activations in the left thalamus, while DD patients had increased activations in the right supramarginal gyrus. In both cases, hyper-activations are likely to reflect compensatory efforts. In addition, SCZ patients had decreased activations in the left superior parietal gyrus compared to both HC and DD patients. This latter result tentatively suggests that the neurophysiologic processes underlying visuospatial abilities are partially preserved in DD, relative to SCZ patients, consistently with the findings showing that cannabis abuse in schizophrenia is associated with better cognitive functioning. Further fMRI studies are required to examine the neural correlates of other cognitive dysfunctions in schizophrenia patients with and without comorbid cannabis use disorder.

## 1. Introduction

Neuropsychological studies have shown that 70% to 75% of patients with schizophrenia have significant cognitive deficits [1]. These deficits encompass attention, reasoning and problem solving, speed of processing, verbal memory, visual memory, and working memory [2]. Cognitive performance of patients with schizophrenia is 1 to 1.5 standard deviations below the performance of the general population [3]. Importantly, cognitive deficits are better predictors of social and occupational functioning than positive and negative symptoms [4].

The cognitive deficits of schizophrenia may be further amplified by the chronic use of psychoactive substances. In schizophrenia, the lifetime prevalence of substance use disorders approaches 50%; this estimate represents a 3- to 5-fold increased risk relative to the general population [5, 6]. Noteworthy, in younger schizophrenia populations, cannabis is one of the most frequently used psychoactive substances with lifetime prevalence rates of cannabis abuse/dependence up to 45% [7]. In addition to producing acute psychotic-like experiences [8], cannabis smoking has been shown to increase the risk for psychotic outcomes in nonpsychosis individuals, independently of intoxication effects [9]. Like other psychoactive substances, cannabis negatively interferes with the course and treatment of schizophrenia. Cannabis abuse/dependence is indeed associated with higher psychotic relapses and hospitalization rates, more severe positive symptoms, nonadherence to antipsychotic therapy, and an earlier age of schizophrenia onset, as well as more suicide attempts [6, 10–12]. In sharp contrast with these findings, the literature investigating cognition has shown that cannabis smoking is associated with *better* cognitive performance in schizophrenia.

In nonpsychiatric smokers, cannabis intoxication has been consistently shown to impair working memory, executive functions, and attention as well as (verbal) episodic memory [13–15]. The residual cognitive effects of cannabis use have also been studied, and the available evidence gathered thus far suggests that the short abstinence from cannabis smoking is associated mostly with deficits in (verbal) episodic memory, executive functions and attention, and possibly visuospatial abilities, while working memory remains largely intact [13, 16, 17]. Theoretically, cannabis smoking should therefore exacerbate the cognitive deficits observed in schizophrenia. However, cross-sectional studies examining this question have shown precisely the reverse, namely, that cannabis smoking/abuse in schizophrenia is associated with fewer deficits in various cognitive domains, including speed of processing, reasoning and problem solving, visual memory, working memory, and visuospatial abilities [18–23], and most of these results have been recently confirmed by a meta-analysis of 10 cognitive studies performed by Yücel et al. [24]. At the moment, the reasons for these seemingly paradoxical findings remain elusive. Nevertheless, most authors in the field consider unlikely that cannabis smoking may actually improve cognitive functioning in schizophrenia [20, 25], and consider, instead, that the better cognitive performance of cannabis smoking schizophrenia patients would be primary, not secondary, to cannabis smoking [22, 24]. According to this perspective, the relatively preserved cognitive performance of dual-diagnosis patients reflects a relatively lower vulnerability for psychosis and a developmental trajectory in which cannabis smoking is required to trigger psychosis [26].

Despite the reliable evidence discussed above, only one functional imaging study (to our knowledge) has examined the neural correlates of cognitive functioning in cannabis smoking patients with schizophrenia. Using an attention task (auditory dichotic listening), this functional magnetic resonance imaging (fMRI) study revealed that schizophrenia patients with previous cannabis use (*n* = 13) had increased activations in the right posterior cingulate cortex, the right inferior parietal lobe, and the right precentral gyrus, relative to the nonusing schizophrenia group (*n* = 13), indicating less impaired brain functioning in the dual-diagnosis group [27]. Here, we sought to further the understanding of the neurophysiological processes underlying the better cognitive performance of schizophrenia patients who smoke cannabis by measuring the patients' cerebral activations while performing a cognitive task. Since schizophrenia patients with substance use disorders are more frequently males than nonusing schizophrenia patients [28], we decided to recruit only male participants. Importantly, mounting evidence suggests that cognitive functioning is influenced by sex differences in schizophrenia [29, 30]. Recently, our group has studied schizophrenia patients' visuospatial abilities, which are known to be impaired in the disorder [23, 31–33] and found that deficits in mental rotation (as well as their neural correlates) between schizophrenia patients and healthy controls were much more pronounced in males than among females [34, 35]. This specific visuospatial task seemed therefore suited to increase the likelihood of detecting significant cognitive and neurophysiologic differences between *male* schizophrenia patients with and without cannabis smoking.

In healthy controls, various fMRI studies have shown that visuospatial abilities, as measured commonly with mental rotation tasks, depend closely on the recruitment of frontal, premotor, thalamic, and parietal regions [36–39]. In schizophrenia, frontal, and parietal abnormalities have been regularly reported in structural imaging studies, and resting-state functional connectivity studies as well as fMRI studies examining the neural correlates of cognitive performance on various visuospatial tasks [40–44]. Based on the current state of knowledge, our a priori hypotheses are that the cannabis abuse/dependence in schizophrenia will be associated with better mental rotation performance as well as increased visuospatial-related activations.

## 2. Material and Methods

### 2.1. Participants

Twenty-eight outpatients meeting DSM-IV criteria for schizophrenia (APA) [45], in a stable phase of illness (no hospitalization within the last two months and no antipsychotic change within the last month) were divided into two groups: 14 patients diagnosed with cannabis use disorder (last 6 months) (dual diagnosis-DD) and 14 patients without substance use disorder (SCZ). We also added 21 healthy controls (HC). Participants were all men; aged between 18 and 55 years; with no concomitant neurological, axis I, or axis II disorders, including schizophreniform or schizoaffective disorders; and no contraindications for functional magnetic resonance imaging (fMRI). Importantly, DD patients did not abuse any other psychoactive substance.

Patients were evaluated by experienced psychiatrists using DSM-IV criteria [45]. Controls were screened with the nonpatient edition of the *Structured Clinical Interview for DSM-IV *[46]. Symptoms severity was rated with the *Positive and Negative Syndrome Scale* [47]. Between-group comparison of antipsychotic dosage was calculated using chlorpromazine equivalents [48]. We assessed the parental socioeconomic status for each participant according to the National Occupational Classification [49]. Participants were required to abstain from smoking cannabis during the day of their scheduled appointment. Prior to being scanned, patients were carefully screened for signs of cannabis intoxication (e.g., impaired motor coordination, conjunctival dilatation, euphoria) or withdrawal (e.g., nervousness, mood swings, headaches, appetite, or sleep disturbances) by a psychiatrist (MD) experienced in drug addiction diagnoses. Participants were also required to fill a self-report questionnaire assessing the frequency of their cannabis consumption. Based on these self-reports, 43% of our sample were completely abstinent from cannabis for more than a month.

In agreement with the *Declaration of Helsinki*, written informed consent was obtained from each participant. The study was approved by the ethics committees of the *Fernand-Seguin Research Center* and the *Réseau de Neuroimagerie du Québec*.

### 2.2. Experimental Procedure

Our version of the mental rotation task consisted of an 8-minute run of alternating 38-second blocks of experimental and control conditions with 20-second periods of rest separating the blocks from one another. Both types of blocks (experimental and control) were repeated four times during the course of the functional run and involved presentations of pairs of 3D shapes, adopted from Shepard and Metzler's [50] mental rotation task. In the experimental condition, one shape was rotated along its vertical axis relative to the other shape. In half of the trials, the figures were identical to each other, whereas in the other half they were mirror images of each other. In the control condition, participants were presented with the un-rotated identical or mirror 3D drawings. In both conditions participants had to determine (by pressing a button with their right index or middle finger) whether the two shapes were identical or mirror images of each other. Each picture appeared for duration of 3 s followed by a blank screen with a fixation point for an average of 1.75 s (ranging from 1 to 2.5 s and giving an average interstimulus interval (ISI) of 4.75 s).

### 2.3. fMRI Data Acquisition

We recorded blood oxygenation level dependent (BOLD) signals using a single-shot, gradient-recalled echo-planar imaging sequence (repetition time (TR) = 3000 ms, echo time (TE) = 30 ms, flip angle = 90°, matrix size = 64 × 64 voxels, and voxels size = 3.5 × 3.5 × 3.5 mm^3^) on a Siemens TRIO MRI system at 3.0 Tesla at the *Functional Neuroimaging Unit at the University of Montreal Geriatric Institute*. We then registered the functional volumes to individual high-resolution coplanar anatomical images taken during the same scanning session (Please refer to [51]).

### 2.4. fMRI Data Analysis

We analyzed fMRI data using a statistical parametric mapping software (SPM5: Wellcome Department of Cognitive Neurology, London, UK) according to the methods outlined by Friston [52]. The functional images were realigned to the mean volume of the run to correct for artifacts due to minor head movements, high-pass filtered, spatially normalized into the standardized brain template, and spatially smoothed with a three-dimensional isotropic Gaussian kernel (8 mm FWHM) to improve signal-to-noise ratio. 

We used a standard peak-detection approach and the general linear model implemented in SPM5 for our statistical analyses in order to identify the dynamic cerebral changes associated with mental rotation. Block design analyses were performed with SPM-5 using a 2-level procedure. At the first level, a separate general linear model was specified for each participant to investigate individual brain activation maps associated with the mental rotation contrast (experimental minus control condition). Second-level random-effects models were then implemented to investigate the pattern of activations during the mental rotation contrast (experimental minus control condition) in each group, using one-sample student's *t*-tests, and between groups, using two-sample student's *t*-tests. Unlike fixed-effects, the random-effects model takes into account intersubject variance permitting population-level inferences [53]. A hypothesis-driven approach was adopted, and region of interest (ROI) analyses were performed, using the “small volume correction” (radius = 12 mm) and *Automated Anatomical Labeling* [54] functions of SPM-5 with a threshold of *P* < 0.05, false discovery rate (FDR) corrected for multiple comparisons. The choice of our ROIs was based on previous fMRI studies on visuospatial abilities [36–39] and included the inferior, middle, and superior frontal gyris, the inferior and superior parietal gyri, premotor regions (precentral gyrus and supplementary motor area), and the thalamus.

### 2.5. Behavioral Data Analyses

To examine between-group differences in sociodemographic, clinical, and cognitive variables, we conducted one-way analyses of variance with diagnosis (HC, DD, and SCZ) as the independent variable. Where we detected group effects, we further investigated the source of these effects by performing multiple comparisons. For dichotomic variables, we performed Pearson's chi-square tests. The level of significance was set at *P* < 0.05. Statistical analyses were performed with the *Statistical Package for the Social Sciences*.

## 3. Results

### 3.1. Demographic and Clinical Data

The three groups were matched for age and handedness. HC were more educated than both groups of patients (*P* = 0.001), but the level of education did not differ between DD and SCZ patients. DD patients had a poorer parental socioeconomic status relative to HC and SCZ patients (*P* = 0.001 and *P* = 0.034, resp.), but socioeconomic status did not differ between HC and SCZ patients. DD presented similar positive, negative, general, and depressive symptoms as SCZ patients. The patient groups had an equivalent age of onset of schizophrenia, had a similar length of illness, and received comparable chlorpromazine equivalents (Table 1).

### 3.2. Cognitive Data

As shown in Table 2, both schizophrenia groups (SCZ and DD) had lower accuracy during the mental rotation task, compared to HC, but no difference emerged between SCZ and DD patients. Similarly, both schizophrenia groups (SCZ and DD) had slower reaction times during the mental rotation task, compared to HC, but no difference emerged between SCZ and DD patients (Table 2).

### 3.3. fMRI Data

#### 3.3.1. One-Sample Student's *t*-Tests for the Mental Rotation Contrast (Experimental Minus Control Condition)

**Figure 1:**
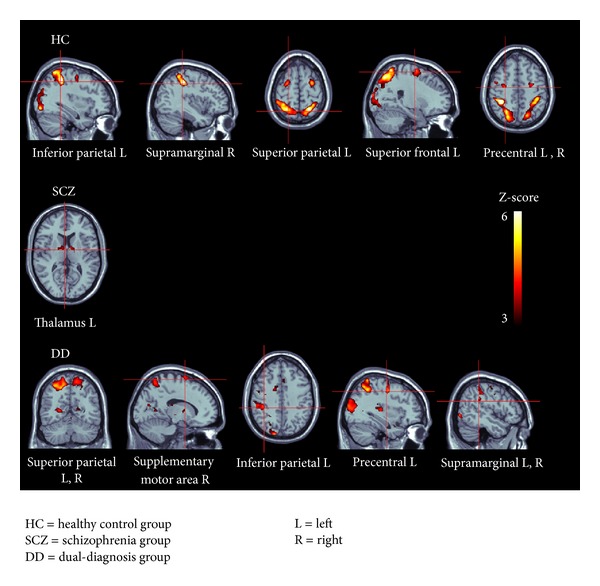
ROI brain activity when processing the mental rotation task in the 3 groups. HC: healthy control group; SCZ = schizophrenia group; DD = dual-diagnosis group; L = left; R = right.

ROI analyses revealed significant loci of activations in the left inferior and superior parietal gyrus, the right supramarginal gyrus, and the left superior frontal gyrus as well as the bilateral precentral gyrus in the HC group. In the SCZ group, we observed significant activations restricted to the left thalamus. Finally, the DD group presented significant loci of activations in the bilateral superior parietal gyrus, the left inferior parietal gyrus, the right supplementary motor area, the left precentral gyrus, and the left and right supramarginal gyrus (Table 3 and Figure 1).

#### 3.3.2. Two-Sample Student's *t*-Test for the Mental Rotation Contrast (Experimental Minus Control Condition)

Between-group analyses revealed increased loci of activations in the left superior parietal cortex in HC relative to the SCZ group (MNI coordinates: *x* = −28; *y* = −60; *z* = 66; 27 voxels; *z* = 3.59; *P* = 0.023). Conversely, we observed increased cerebral activations in the left thalamus in SCZ compared to HC (MNI coordinates: *x* = −10; *y* = −4; *z* = 7; 30 voxels; *z* = 3.31; *P* = 0.050). When looking at the comparison between HC and DD groups, we found that HC did not show any increased activations relative to DD, while DD presented significantly more activations in the right supramarginal gyrus (MNI coordinates: *x* = 63; *y* = −38; *z* = 24; 37 voxels; *z* = 3.55; *P* = 0.025). Finally, relative to the SCZ group, DD showed increased activations in the left superior parietal gyrus (MNI coordinates: *x* = −32; *y* = −52; *z* = 70; 28 voxels; *z* = 3.31; *P* = 0.001). In contrast, no significantly increased loci of activations were observed in the SCZ group, relative to the DD group.

## 4. Discussion

In view of the literature showing that cannabis smoking/abuse is associated in schizophrenia with better performance in various cognitive domains, including visuospatial abilities [18, 19, 21, 23, 24, 55, 56], we sought to examine the neural correlates of mental rotation in schizophrenia patients with and without cannabis abuse/dependence. We found that both schizophrenia groups performed more poorly than controls on a mental rotation task, a result consistent with the extensive cognitive literature showing that schizophrenia patients have impaired visuospatial abilities [31–33]. In contrast, we found no differences in mental rotation performance between DD and SCZ patients. Neurally, we found that HC activated various frontal (superior frontal and precentral gyri) and parietal regions (inferior and superior parietal as well as supramarginal gyri) and that DD patients (but not SCZ patients) activated similar frontal (precentral gyrus and supplementary motor area) and parietal regions (inferior and superior parietal as well as supramarginal gyri). As such, these results are highly consistent with the fMRI literature showing that the frontal and parietal lobes play a key role in the processing of visuospatial abilities [36, 38, 57, 58]. Noteworthy, while brain activations in precentral regions and motor areas (e.g., supplementary motor area) during mental rotation may be induced by eye movements [57], several studies have underlined, on the contrary, the direct involvement of those regions in mental rotation processes [59–63]. 

More importantly, our between-group comparisons revealed increased activations in the left superior parietal gyrus in both HC and DD, relative to SCZ patients, while the comparison between DD patients and HC revealed no between-group differences regarding brain region (despite the fact that DD patients had lower parental SES status). In fMRI studies examining the neural processes underlying mental rotation and visuospatial processing in healthy subjects, the superior parietal gyrus is one of the regions that have been most consistently activated [38, 58, 64–66]. Moreover, this result is of interest given that parametric studies of brain activity as a function of proportion of rotated stimuli or the rotation angle have observed graded effects specifically in the superior parietal cortex, suggesting that it is the core region of spatial manipulations [36, 67]. Finally, we found that SCZ patients overactivated the left thalamus and that DD patients overactivated the right supramarginal gyrus, compared to HC. These overactivations may reflect compensatory neural responses to an impaired cognitive performance. Interestingly, there is mounting evidence from post mortem, structural, and functional imaging studies showing that the thalamus is prominently impaired in schizophrenia and potentially responsible for the poor coordination of information flow associated with the disorder [68, 69]. As for the supramarginal gyrus, it is a region regularly activated in mental rotation tasks, and it appears to be responsible for space perception and detection of salient stimuli and to also play a role in spatial manipulation but to a lesser extent than the superior parietal cortex [36, 70]. 

To the best of our knowledge, our study is only the second one to examine the neural correlates of cognitive functioning in schizophrenia patients with comorbid cannabis use/abuse using functional imaging. Indeed, in a recent fMRI study, Løberg et al. [27] showed that past cannabis use is associated with increased activations in precentral, cingulate, and parietal regions in schizophrenia patients performing an attention task (e.g., auditory dichotic listening). Unfortunately, this study did not include a control group of HC, making it difficult to determine if cannabis use was truly associated with “normal” activations or if it was actually associated with hyperactivations. Here, in our study, the increased left superior parietal brain activations during mental rotation in DD relative to SCZ patients despite similar cognitive performance does not suggest an inefficient cognitive processing in DD. In effect, the inclusion of a group of HC makes it possible to infer that, during a mental rotation task, DD patients displayed a slightly more “typical” pattern of brain activations, compared to SCZ patients, although both schizophrenia groups had a similarly impaired cognitive performance.

Our results need to be discussed cautiously. On one hand, the finding of a spared functioning of the left superior parietal gyrus in DD relative to SCZ patients tentatively suggests that cannabis has neuroprotective effects in schizophrenia. Cannabis produces its effects on the brain via the endogenous cannabinoid system, which is composed of (at least) two principal ligands, anandamide, and 2-arachidonoylglycerol, which bind (at least) two cannabinoid receptors (CB_1_ and CB_2_) [71, 72]. Given that endocannabinoids have been shown to exert neuro-protective effects in animals via immune-modulatory mechanisms, microglial activation, and/or protection against excito-toxicity [73], chronic cannabis smoking may normalize the neural processing of cognition (here, mental rotation) in schizophrenia. However, this interpretation is unlikely for 4 main reasons. First, the acute administration of delta-9-tetrahydrocannabinol (Δ^9^-THC, the main psychoactive agent of cannabis) to healthy controls has been shown to impair cognition, mostly attention, episodic memory, working memory, and executive functions [14, 15]. Similarly, the residual effects of chronic cannabis smoking have been linked with impairments in executive functions, attention, and episodic memory, as well as small and inconsistent impairments in visuospatial abilities [16, 17, 74]. Second, functional imaging studies performed in healthy volunteers revealed only inconsistent and contradictory effects of acute Δ^9^-THC (or marijuana) administration on parietal functioning [75–77]. Similarly, functional imaging studies on the residual effects of cannabis in resting state or task-related conditions did not evidence marked and unequivocal changes in the functioning of parietal regions [75, 78, 79]. Third, Δ^9^-THC has been intravenously administered by D'Souza et al. [80] to schizophrenia patients and HC, and the authors found that Δ^9^-THC aggravated the positive and negative symptoms of schizophrenia and impaired cognition in both groups. Finally, structural MRI and diffusion-tensor imaging studies have not linked cannabis smoking with clearly protective effects in schizophrenia, as studies have produced conflicting evidence of increased [81, 82], equivalent [83, 84] or decreased neuroanatomic alterations [85].

As an alternative to the above-mentioned neuro-protection hypothesis, it has been proposed that in order to sustain the lifestyle of substance abuse (make deals, find money, etc.), patients with schizophrenia have to be able to maintain minimal social contacts and apply at least some organizational strategies. According to this view, one would expect substance-abusing schizophrenia patients to have relatively spared cognitive abilities [86, 87]. This second interpretation, however, has recently been put in doubt by Schnell et al. [22], because cannabis has become an easily accessible substance in the west and acquiring it no longer calls for elaborate relational or cognitive aptitudes. As proposed by Schnell et al. [22], we may equally interpret this finding of relatively better cognitive function (and/or better neural processing) among patients with comorbid schizophrenia and cannabis abuse in light of the literature showing that cannabis smoking may be a risk factor for psychotic symptom development [9]. This association may imply that schizophrenia would have not developed in patients with dual disorders had they not compulsively smoked cannabis. We would therefore expect these patients to have milder deficits on key phenotypic characteristics of schizophrenia, including cognitive dysfunctions and their neural underpinnings. Here, DD patients did not perform better cognitively than SCZ patients on the mental rotation task, as in the Løberg et al. study [27], most probably due to lack of statistical power in both studies. The more typical pattern of brain activations in the DD group may nevertheless reflect a relatively lower vulnerability for psychosis [22].

Our study comprised a few limitations. First, we did not scan a group of nonpsychosis patients with cannabis abuse/dependence. The inclusion of such a group may have clarified aspects of our results. However, here, we found that cannabis abuse/dependence was associated with spared superior parietal functioning in schizophrenia, whereas chronic smoking is known to impair, not to improve, the neurophysiologic processes underlying various cognitive functions in otherwise healthy subjects [88]. Second, our DD group included only 14 patients. However, this population is typically noncompliant to treatment and therefore difficult to scan. We paid great attention to our recruitment method in order to ensure that DD patients did not suffer from cannabis-induced psychosis and did not abuse any other psychoactive substances. Finally, it may have been relevant to investigate a cognitive domain (e.g., verbal memory, for instance) known to be more significantly impaired by chronic cannabis smoking than visuospatial abilities.

## 5. Conclusions

Together with the literature on cognition, the results of the current fMRI study provide preliminary evidence that some cognitive-related neurophysiologic processes are partially spared in cannabis-smoking schizophrenia patients. Future fMRI studies in the field will need to examine the neural correlates of cognitive functions other than visuospatial abilities in larger sample of patients. Studies will also need to determine whether our results hold true for female DD patients. Finally, longitudinal studies will need to be performed in order to find out if the relatively preserved superior parietal functioning of DD patients is primary or secondary to cannabis smoking. Such studies would measure cognition and their neural correlates when patients are in the active smoking phase and after prolonged abstinence. Studies initiated during the prodromal phase of psychosis are also warranted. 

## Figures and Tables

**Table 1:** Sociodemographic and clinical data.

	Control group (*N* = 21)	SCZ group (*N* = 14)	DD group (*N* = 14)
Age (years)	30.3 (7.9)	32.6 (8.4)	30.9 (11.5)
Lefthanded	4	4	1
Education level (years)	18.0 (2.9)**	11.1 (2.9)	10.0 (1.9)
Parental SES	2.4 (1.1)**	2.9 (0.7)*	3.8 (1.2)
Clinical			
Age of onset (years)	—	20.8 (4.6)	20.3 (4.4)
Duration of illness (years)	—	11.9 (9.0)	10.6 (12.2)
Total medication (mg/day)	—	568 (276)	553 (392)
PANSS positive	—	17.1 (4.7)	16.4 (6.0)
PANSS negative	—	19.9 (6.2)	18.9 (5.3)
PANSS general	—	37.3 (5.2)	32.5 (8.7)
Calgary Depression Scale	—	3.7 (2.1)	3.8 (4.1)
Antipsychotics	—	risperidone (6), quetiapine (3), olanzapine (6), clozapine (5)	risperidone (7), quetiapine (4), olanzapine (3), clozapine (1), typical (2)

DD: dualdiagnosis; SCZ: schizophrenia; SES: socioeconomic status; total medication is in chlorpromazine equivalents; (SD in parentheses); *P < 0.05; **P = 0.001.

**Table 2:** Mental rotation performance in schizophrenia and dual-diagnosis patients and healthy controls.

Score	Control group (*N* = 21)	SCZ group (*N* = 14)	DD group (*N* = 14)	Statistics	Multiple comparisons*
Accuracy (%)	94.6 (5.5)	76.7 (11.0)	72.4 (14.4)	*F* = 23.4; *P* = 0.0001	Controls > SCZ & DD
Reaction time (s)	1.5 (0.4)	2.0 (0.2)	1.9 (0.4)	*F* = 9.0; *P* = 0.001	Controls < SCZ & DD

DD: dualdiagnosis; SCZ: schizophrenia; *multiple comparisons without Bonferroni correction.

**Table 3:** ROI activations during the mental rotation task (experimental relative to control condition) in the 3 groups (1-sample student's *t*-test).

Brain region	R/L	MNI coordinates			*Z*-score	Voxels	*P* value
		*x*	*y*	*z*			
Control group							
Inferior parietal	L	−35	−42	46	5.30	120	0.001
Supramarginal	R	38	−35	42	5.14	86	0.001
Superior parietal	L	−24	−60	63	4.97	169	0.001
Precentral	L	−28	−14	49	4.32	36	0.003
Superior frontal	L	−24	−7	63	4.00	43	0.003
Precentral	R	32	−7	56	4.30	80	0.001
SCZ group							
Thalamus	L	−7	−4	10	3.37	31	0.050
DD group							
Superior parietal	L	−18	−63	49	3.97	198	0.002
Supplementary motor area	R	14	4	70	3.71	17	0.013
Precentral	L	−35	−10	46	3.64	27	0.012
Inferior parietal	L	−38	−35	38	3.45	27	0.010
Supramarginal	L	−49	−38	28	3.42	19	0.018
	R	56	−28	52	3.21	15	0.036
Superior parietal	R	21	−56	56	3.44	43	0.008

R: right; L: left; *P* is FDR corrected at 0.05.
